# Renoprotective Effects of Atorvastatin in Diabetic Mice: Downregulation of RhoA and Upregulation of Akt/GSK3

**DOI:** 10.1371/journal.pone.0162731

**Published:** 2016-09-20

**Authors:** Thiago Bruder-Nascimento, Glaucia Callera, Augusto Cesar Montezano, Tayze T. Antunes, Ying He, Aurelie Nguyen Dinh Cat, Nathanne S. Ferreira, Pedro A. Barreto, Vânia C. Olivon, Rita C. Tostes, Rhian M. Touyz

**Affiliations:** 1 Department of Pharmacology, Ribeirao Preto Medical School, University of Sao Paulo, Ribeirao Preto, Brazil; 2 Kidney Research Centre, Ottawa Hospital Research Institute, University of Ottawa, Ottawa, Canada; 3 Institute of Cardiovascular and Medical Sciences, University of Glasgow, Glasgow, United Kingdom; Max Delbruck Centrum fur Molekulare Medizin Berlin Buch, GERMANY

## Abstract

Potential benefits of statins in the treatment of chronic kidney disease beyond lipid-lowering effects have been described. However, molecular mechanisms involved in renoprotective actions of statins have not been fully elucidated. We questioned whether statins influence development of diabetic nephropathy through reactive oxygen species, RhoA and Akt/GSK3 pathway, known to be important in renal pathology. Diabetic mice (db/db) and their control counterparts (db/+) were treated with atorvastatin (10 mg/Kg/day, p.o., for 2 weeks). Diabetes-associated renal injury was characterized by albuminuria (albumin:creatinine ratio, db/+: 3.2 ± 0.6 *vs*. db/db: 12.5 ± 3.1*; *P<0.05), increased glomerular/mesangial surface area, and kidney hypertrophy. Renal injury was attenuated in atorvastatin-treated db/db mice. Increased ROS generation in the renal cortex of db/db mice was also inhibited by atorvastatin. ERK1/2 phosphorylation was increased in the renal cortex of db/db mice. Increased renal expression of Nox4 and proliferating cell nuclear antigen, observed in db/db mice, were abrogated by statin treatment. Atorvastatin also upregulated Akt/GSK3β phosphorylation in the renal cortex of db/db mice. Our findings suggest that atorvastatin attenuates diabetes-associated renal injury by reducing ROS generation, RhoA activity and normalizing Akt/GSK3β signaling pathways. The present study provides some new insights into molecular mechanisms whereby statins may protect against renal injury in diabetes.

## Introduction

Kidney disease is associated with end-stage renal disease and continues to be a major cause of morbidity and mortality in patients with diabetes [1–3]. Several factors contribute to the progression of diabetic nephropathy, including hyperglycemia, hypertension, dyslipidemia, production of inflammatory cytokines and oxidative stress, triggering hypertrophic, proliferative, inflammatory and apoptotic events [2–4]. Identifying targets to ameliorate these processes may be renoprotective and thereby reduce the renal complications of diabetes. While many therapeutic approaches have been considered, there is growing interest in the potential beneficial effect of statins.

Statins are competitive inhibitors of 3-hydroxy-3-methylglutaryl coenzyme A (HMG-CoA) reductase, a major enzyme in cholesterol synthesis, thus decreasing endogenous cholesterol formation [5]. Clinical benefits of cholesterol reduction have been established in primary and secondary interventional studies using statins, demonstrating that treatment with these drugs decreases morbidity and mortality related to coronary heart disease [6, 7]. In addition, statins have recently emerged as novel renoprotective drugs in several models of kidney injury, including diabetic nephropathy, and for this reason are being investigated in a number of clinical trials [8–10].

The protective effects of statins in the cardiovascular and renal systems may occur independently of their lipid-lowering effect, and are referred as pleiotropic effects. Inhibition of activity of small GTPases [small hydrolase enzymes that can bind and hydrolyze guanosine triphosphate (GTP)] is one of the major pleiotropic actions of statins [11–13]. These small G proteins, including Ras, Rho, Rab, and Ran, are monomeric proteins with a low molecular mass that elicit various key intracellular signals. In particular, a series of recent investigations has revealed a novel contribution of Rho and its effector molecule, Rho-kinase, to renal disease via inhibition and/or activation of different signaling pathways, such as Akt and NADPH oxidase, leading to modulation of cell motility, apoptosis and proliferative responses. However, it remains unclear whether RhoA contributes to renal hypertrophy in diabetic individuals [2, 14–17].

Considering that some of the beneficial effects of statins are mediated by antioxidant effects and RhoA inhibition [16, 18], the aim of the present study was to determine whether atorvastatin decreases RhoA activity, ROS signaling and associated renal damage in the leptin receptor deficient db/db mouse, a rodent model of obesity and type 2 diabetes. In addition, we questioned whether the Akt/GSK3β pathway, important in cell survival, is sensitive to effects of atorvastatin. We tested the hypothesis that atorvastatin, through redox-sensitive and RhoA-dependent mechanisms, attenuates diabetes-associated nephropathy. We also questioned whether atorvastatin prevents renal hypertrophy by modulating the Akt/GSK3β pathway. We previously reported that atorvastatin ameliorates vascular injury in db/db mice. Here we extend that study by investigating effects of atorvastatin on diabetic nephropathy. To respect the principles of the 3Rs (Replacement, Reduction and Refinement) in research, we examined mice that we have previously characterized [19].

## Material and Methods

### Animals

The study was approved by the Animal Ethics Committee of Ottawa Hospital Research Institute—University of Ottawa. Experiments were conducted in accordance with the guidelines from the National Institutes of Health Guide for the Care and Use of Laboratory Animals and with Institutional guidelines. Leprdb/Lepr+ (db/+) and Leprdb/Leprdb (db/db) mice (B6.BKS (D)-Leprdb/J) were purchased from Jackson Laboratories (Maine, USA). Male mice (8 weeks of age) were treated with 10 mg/kg/day atorvastatin (Millipore Corporation, Billerica, MA, USA), p.o. for 2. Atorvastatin was incorporated into the chow (2018 Teklad Global 18% Protein Rodent Diet, Harlan Laboratories, Madison, WI), after previous assessment of daily food intake (db/+: 0,063 g atorvastatin/Kg chow; db/db: 0,056 g atorvastatin/Kg chow). Mice were killed by CO_2_ inhalation. Kidney samples used in this study were obtained from mice used in our previous study [19].

### Assessment of urinary albumin and creatinine

Spot urine samples were collected immediately before death. Urine albumin-to-creatinine ratio was measured using a commercial kit (Albuwell and Creatinine companion kit; Exocell, Philadelphia, PA) as recommended by the Animal Models of Diabetic Complications Consortium [20].

### Renal hypertrophy and histopathologic analysis

Kidneys were removed and weighed. The kidney weight (g) was corrected by the length of the tibia (mm). Kidneys were fixed in 4% paraformaldehyde (PFA) solution for 24 h, dehydrated, embedded in paraffin and transversely sectioned (4 μm). Sections were stained with Masson Trichrome for measurement of mesangial (MA) and glomerular (GA) areas. Sections were visualized with a Zeiss AxioCam HRc (Zeiss Axio Imager; Zeiss, Oberkochen, Germany), and a magnification of 100x. At least 20 glomeruli in each section (two sections per kidney) from five mice/group were examined. Glomerular area was measured using Masson Trichrome-stained kidney sections (ImageJ software, National Institute of Health).

### Detection of renal NADPH oxidase activity by lucigenin chemiluminescence

Activation of renal NADPH oxidase was assessed using the lucigenin-derived chemiluminescence assay [21].

### Immunohistochemistry

Localization of PCNA and RhoA in kidneys was studied by immunohistochemistry using pol- yclonal antibodies against anti-proliferating cell nuclear antigen (PCNA) (Cell Signaling, Danvers, Massachusetts, USA) and RhoA (Abcam, Cambridge, MA, USA). Four-μm thick sections of ZBF-fixed, paraffin-embedded kidneys on Superfrost Plus slides were deparaffinised in xylene and rehydrated in graded alcohol baths. Immunohistostaining was performed according to the manufacturer’s instructions (Vector Laboratories, Burlingame, CA, USA).

### Immunoblotting

Total protein was extracted from the kidneys cortex. Briefly, frozen tissues were homogenized in 50 mM Tris-HCl (pH 7.4) lysis buffer (containing 1% Nonited P-40, 0.5% sodium deoxycholate, 150 mM NaCl, 1 mM EDTA, 0.1% sodium dodecyl sulfate (SDS), 2 mM sodium orthovanadate (Na_3_VO_4_), 1 mM phenylmethylsulfonyl fluoride (PMSF), 1 μg/mL pepstatin A, 1 μg/mL leupeptin and 1 μg/mL aprotinin). Total protein extracts were centrifuged at 10,000 rpm for 10 min and the pellet was discarded. Proteins from homogenates of renal tissues (50 μg) were separated by electrophoresis on a polyacrylamide gel (10%), and transferred onto a nitrocellulose membrane. Nonspecific binding sites were blocked with 5% skim milk or 1% bovine serum albumin (BSA) in Tris-buffered saline solution with Tween for 1 hour at 24°C. Membranes were then incubated with specific antibodies overnight at 4°C. Antibodies were as follows: anti-Nox4, anti-vascular cell adhesion molecule 1 (VCAM-1), anti-osteopontin (OPN), anti-PCNA, anti-glycogen synthase kinase-3 beta (GSK-3β) (Ser^9^); anti-nuclear factor-kB (NF-kB) subunit p65 (Ser^536^), anti-Akt (Ser^473^), anti-p38MAPK (Thr^180^/Tyr^182^), anti- SAPK/JNK MAPK (Thr^183^/Tyr^185^) and anti-ERK1/2 (Thr^202^/Tyr^204^) (Cell Signaling, Danvers, Massachusetts, USA), anti-β-actin (Sigma). After incubation with secondary antibodies, signals were revealed by chemiluminescence, visualized by autoradiography and quantified densitometrically. Values were corrected and normalized for β-actin expression [19, 22].

### Cytosol and membrane fractionation

RhoA activity was determined by measuring RhoA translocation from the cytosol to the membrane. This is a well defined and accepted assay of RhoA activation [8, 23, 24]. Kidney cortex was homogenized in 50 mM Tris-HCl, pH 7.4, lysis buffer containing 5 mmol/L EGTA, 2 mmol/L EDTA, 0.1 mmol/L PMSF, 0.2 mmol/L Na_3_VO_4_, 1 μmol/L pepstatin A, 1 μmol/L leupeptin, and 1 μmol/L aprotinin). Homogenates were centrifuged at 100,000 g for 1 hour at 4°C. The supernatant (cytosolic fraction) was collected. The pellet, containing the particulate fraction, was resuspended in lysis buffer containing 1% Triton X-100 and centrifuged at 10,000 g for 10 min at 4°C. The resultant supernatant was collected (membrane-enriched fraction). Protein analysis was performed by western blotting as described above using anti-RhoA (1:500, Santa Cruz). Antibody to β-actin (Sigma) was used as internal housekeeping control. Results are expressed as the membrane to cytosol ratio of protein content in the cell fractions.

### Data Analysis

Results are presented as mean ± SEM. Comparisons were performed by one-way ANOVA followed by the Bonferroni test or the Newman–Keuls test, when appropriate. P<0.05 was considered statistically significant.

## Results

### Atorvastatin is renoprotective in db/db mice

**Fig 1:**
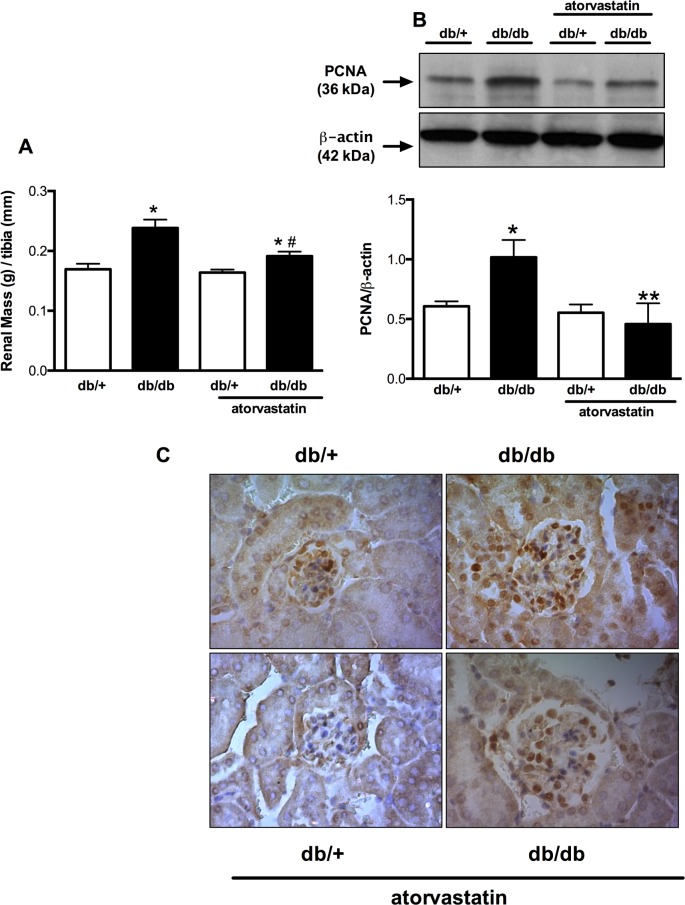
Increased renal mass and PCNA protein content in db/db mice are attenuated by atorvastatin treatment. Renal mass (A) expressed as kidney weight (g) and length of tibia (mm) ratio. PCNA expression (B) and localization (c) determined in the kidney cortex by western blot and immunochemistry, respectively, of db/+ and db/db mice treated with atorvastatin (10 mg/kg/day for 2 weeks) or control diet. Top panel, representative immunoblots of PCNA and β-actin expression. Results are mean ± SEM of 3–6 mice in each experimental group. *P< 0.05 vs. db/+ control diet, **P<0.05 db/db atorvastatin diet vs. db/db control diet and db/+ atorvastatin diet.

Db/db mice exhibited renal hypertrophy and increased PCNA protein expression in tubular epithelial and glomerular mesangial cells in the renal cortex when compared to db/+ mice. Kidney mass was reduced in treated db/db mice versus untreated mice. This was associated with decreased PCNA overexpression (Fig 1A–1C).

**Fig 2:**
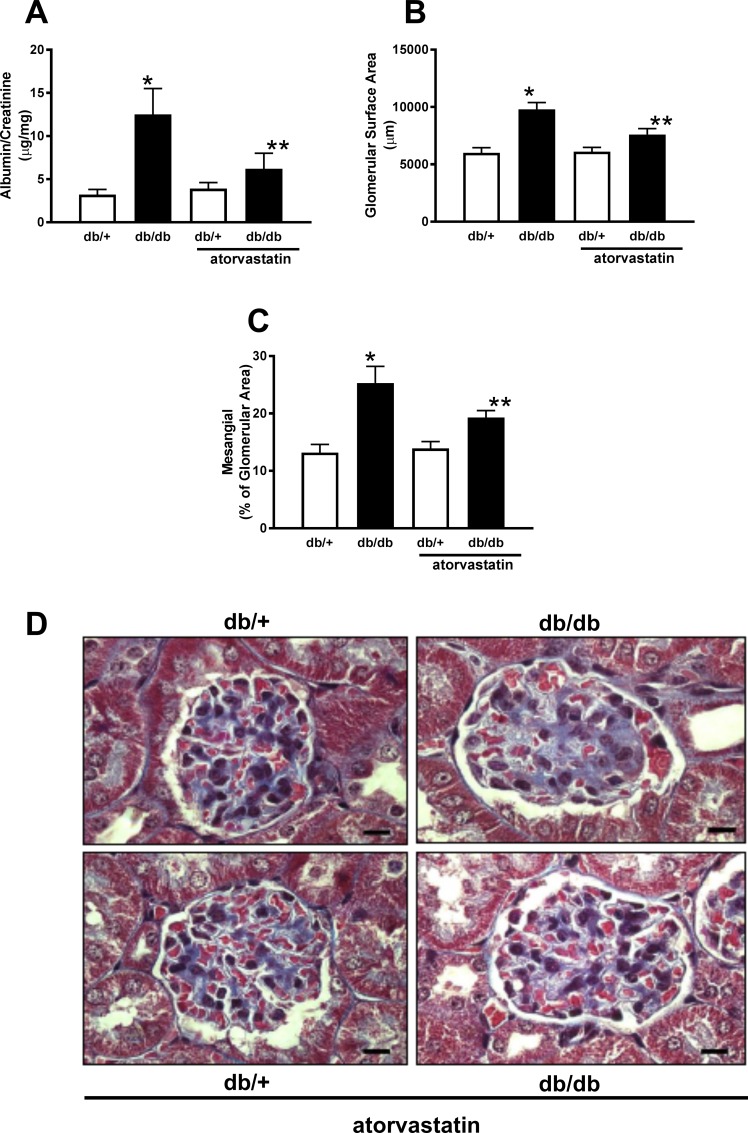
Atorvastatin treatment reduces mesangial expansion in db/db mice. Graphs depict albuminuria (A), glomerular surface area (B), mesangial area, in blue (C) and representative images of a single glomerulus stained with Masson Trichrome (D) from db/+ and db/db mice treated with atorvastatin (10 mg/kg/day for 2 weeks) or control diet. Results are presented as mean ± SEM of 20 glomerular and mesangial areas in sections from 5 mice in each group. *P< 0.05 vs. db/+ control diet, **P<0.05 vs. db/db atorvastatin diet vs. db/db control diet and db/+ atorvastatin diet. (Black scale bar: 10 μm, magnification x100).

Diabetic db/db mice exhibited proteinuria compared to db/+ control mice. Treatment reduced proteinuria in db/db mice (Fig 2A). These functional changes were associated with glomerular structural changes in db/db mice, evidenced by enhanced mesangial (MA) and glomerular (GA) surface areas compared to control db/+ mice. These effects were attenuated in the treated diabetic group (Fig 2B–2D).

### Mechanisms whereby atorvastatin influences renal function in db/db mice–role of RhoA activation

**Fig 3:**
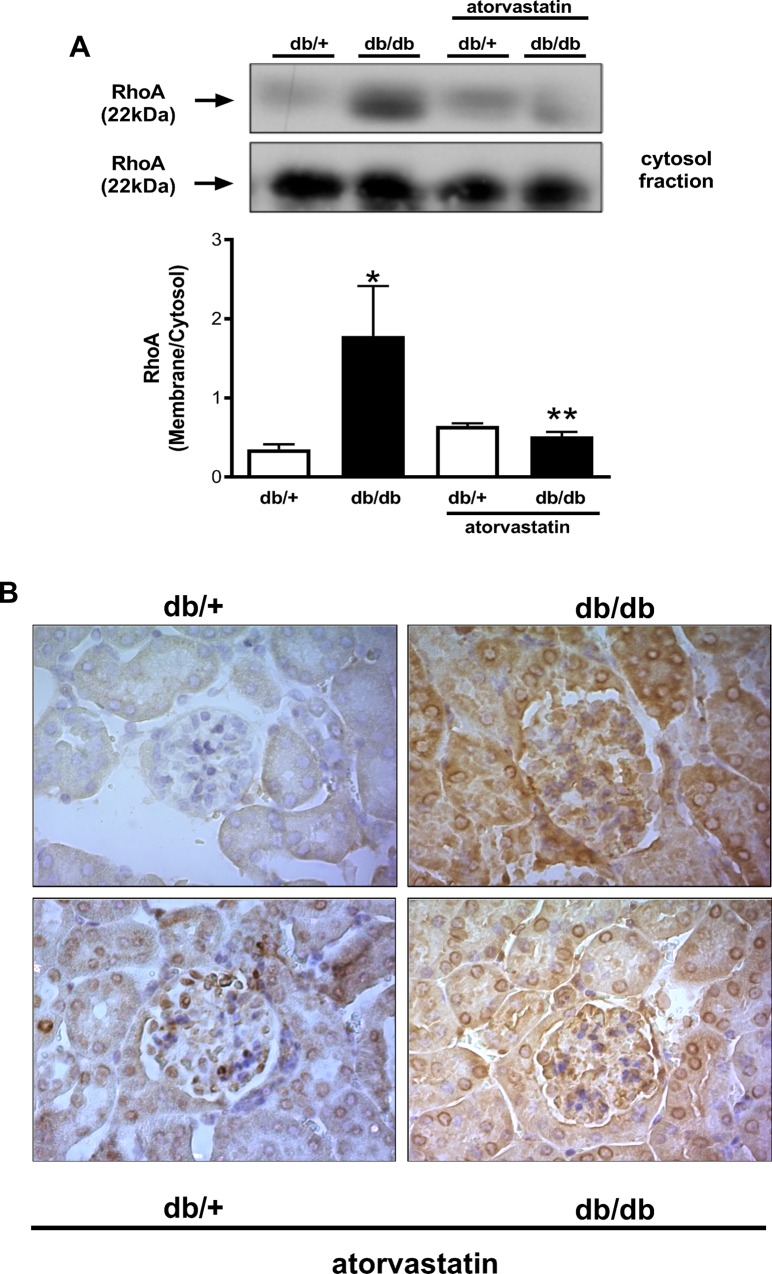
Atorvastatin reduces RhoA translocation from cytosol to the membane in kidneys of diabetic mice. RhoA translocation (A) and RhoA immunolocalization (B) from renal cortex obtained from db/+ and db/db mice treated with atorvastatin (10 mg/kg/day for 2 weeks) or control diet. Translocation of RhoA was assessed by protein expression in the membrane and cytosolic fractions isolated from renal cortex homogenates. Results are mean ± SEM of 3–6 mice in each experimental group. *P< 0.05 vs. db/+ control diet, **P<0.05 db/db atorvastatin diet vs. db/db control diet.

Statins have direct effects on the activity of small GTPases such as RhoA. RhoA activity was assessed by evaluating cytosol-to-membrane translocation of RhoA. Increased RhoA protein content was observed in membrane-enriched fractions of the renal cortex of db/db mice compared with control db/+ mice. The higher membrane:cytosol RhoA expression ratio indicates increased RhoA activity in the renal cortex of the db/db mice. In db/db mice treated with atorvastatin renal RhoA translocation was significantly reduced (Fig 3A). Immunohistochemical analysis revealed that RhoA expression is increased in tubular epithelial cells, as well as in glomerular mesangial cells in renal cortex of db/db mice, versus controls, an effect that is attenuated by atorvastatin (Fig 3B).

**Fig 4:**
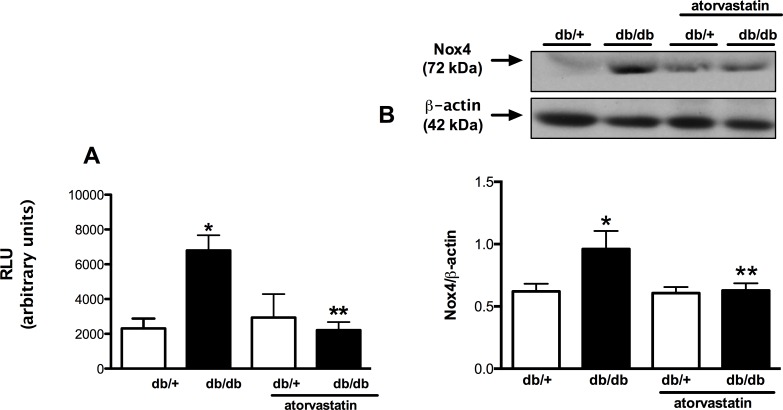
Atorvastatin reduces oxidative stress and Nox4 protein expression in kidney from db/db mice. ROS status was assessed by lucigenin-enhanced chemiluminescence (A) and Nox4 protein expression (B) by western blotting in renal cortex homogenates from db/+ and db/db mice treated with atorvastatin (10 mg/kg/day for 2 weeks) or control diet. Results are mean ± SEM of 6–8 mice in each experimental group. *P< 0.05 vs. db/+ control diet, **P<0.05 db/db atorvastatin diet vs. db/db control diet.

Since oxidative stress triggers various pro-inflammatory, pro-fibrotic and proliferative signaling pathways involved in cardiovascular and renal damage, we analyzed renal NADPH oxidase activity by the lucigenin chemiluminescence assay. Fig 4A shows that diabetic db/db mice exhibited increased superoxide generation in the renal cortex compared to control db/+ mice. This was associated with an increased expression of Nox4 in the renal cortex (Fig 4B). Activation of NADPH oxidase and upregulation of Nox4 were attenuated in treated mice.

### Renal inflammation in diabetic mice is unaffected by atorvastatin

**Fig 5:**
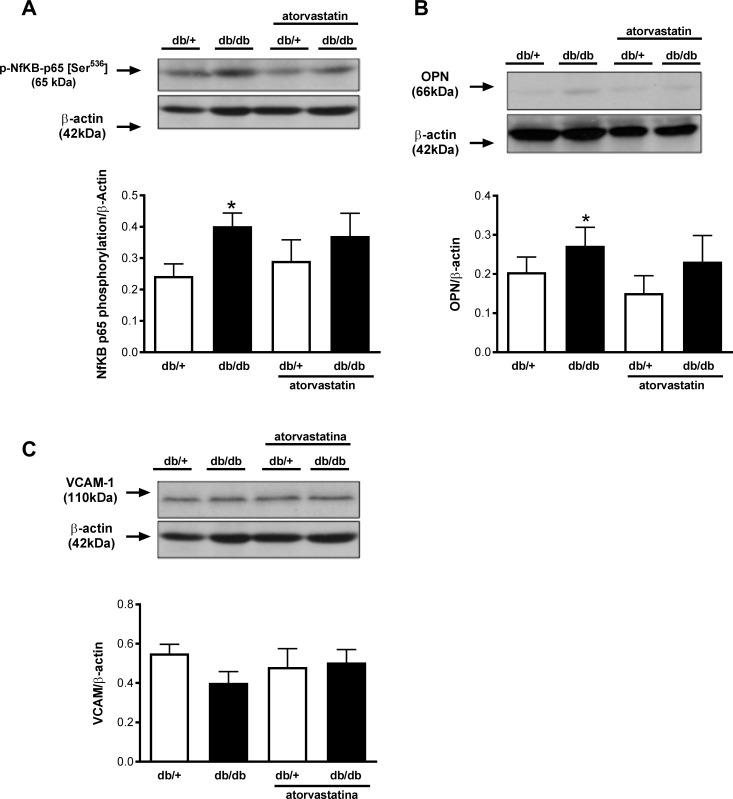
Atorvastatin does not change expression of inflammatory markers in the renal cortex of db/db mice. Phosphorylation of NF-κB p65 (A) and the expression of OPN (B) and VCAM-1 (C) were evaluated in renal cortex isolated from db/+ and db/db mice treated with atorvastatin (10 mg/kg/day for 2 weeks) or control diet. Top panels, representative immunoblots of NF-κB p65 [Ser^536^], OPN, VCAM-1 and β-actin. Results are mean ± SEM of 6–8 mice in each experimental group. *, *P*< 0.05 *vs*. db/+ control diet.

Renal cortex from diabetic mice exhibited increased OPN protein expression and increased phosphorylation of p65 NFkB subunit compared to kidneys from control db/+ mice. Atorvastatin did not influence OPN expression or the magnitude of phosphorylation of p65 NFkB (Fig 5A and 5B). In addition, no differences were observed for VCAM-1 expression between the groups (Fig 5C).

### Differential activation of renal JNK, p38MAPK and ERK1/2 in db/db mice–effects of atorvastatin

**Fig 6:**
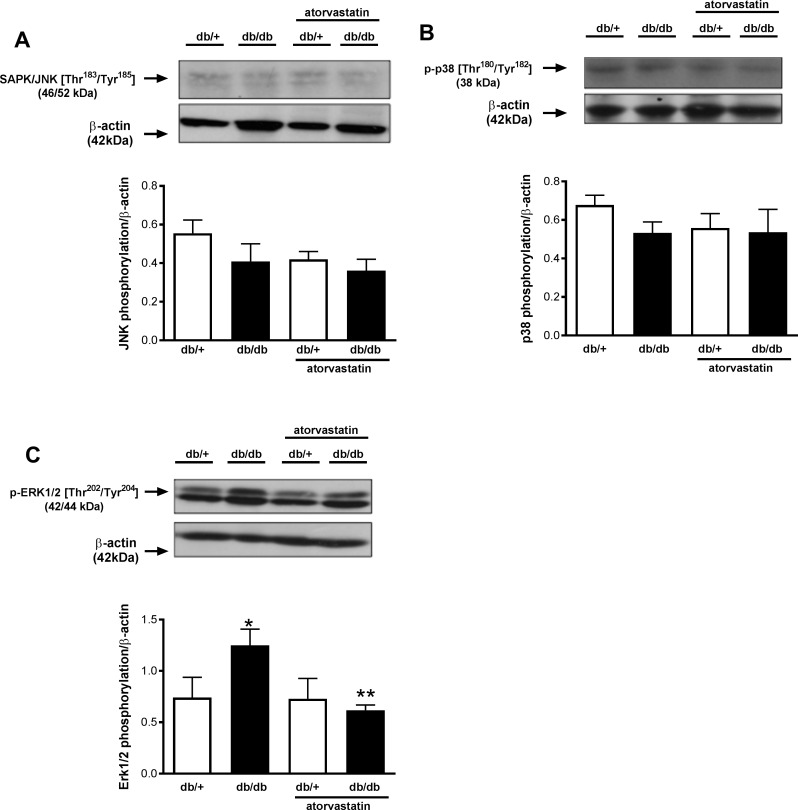
Increased renal ERK1/2 MAPK phosphorylation is abrogated by atorvastatin in db/db mice. Phosphorylation levels of SAPK/JNK MAPK (A), p38 MAPK (B), and ERK1/2 MAPK (C) were evaluated in renal cortex isolated from db/+ and db/db mice treated with atorvastatin (10 mg/kg/day for 2 weeks) or control diet. Top panels, representative immunoblots of SAPK/JNK MAPK [Thr^183^/Tyr^185^], p38 MAPK [Thr^180^/Tyr^182^], ERK1/2 MAPK [Thr^202^/Tyr^204^] and β-actin. Results are presented as mean ± SEM of 6–8 mice in each experimental group. *P< 0.05 vs. db/+ control diet, **P<0.05 db/db atorvastatin diet vs. db/db control diet.

Mitogen-activated protein kinases (MAPK) are redox-sensitive proteins. Since increased ROS generation was detected in the renal cortex of db/db mice, MAPK activation, inferred from phosphorylation levels, was determined in kidneys of diabetic mice by western blot. No differences were observed in SAPK/JNK MAPK or p38 MAPK phosphorylation between the groups (Fig 6A and 6B). ERK1/2 phosphorylation/activity was increased in the renal cortex of diabetic mice, and this was blunted by atorvastatin treatment (Fig 6C). Atorvastatin had no significant effect on phosphorylation of SAPK/JNK MAPK or p38 MAPK.

### Atorvastatin normalizes phosphorylation of Akt and GSK3β in renal cortex of diabetic mice

**Fig 7:**
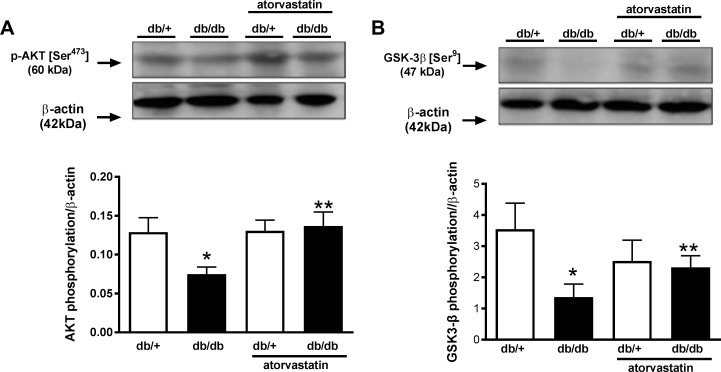
Reduced Akt and GSK3β phosphorylation in the renal cortex of db/db mice is restored by atorvastatin. Phosphorylation levels of Akt (A) and GSK3β (B) were evaluated in renal cortex isolated from db/+ and db/db mice treated with atorvastatin (10 mg/kg/day for 2 weeks) or control diet. Top panels, representative immunoblots of Akt [Ser^473^], GSK3β [Ser^9^] and β-actin. Results are mean ± SEM of 6–8 mice in each experimental group. *P< 0.05 vs. db/+ control diet, **P<0.05 db/db atorvastatin diet vs. db/db control diet.

Renal mass and expression of the proliferative marker PCNA were augmented in renal cortex of diabetic mice. Therefore, the activity of proteins involved in hypertrophic gene programming, specifically Akt and GSK3β, was also investigated. Phosphorylation levels of Akt and GSK3β were significantly reduced in renal cortex of db/db mice compared with control db/+ mice. Atorvastatin restored renal phosphorylation levels of these proteins in db/db mice (Fig 7A and 7B).

## Discussion

Obesity and diabetes are major co-morbidities associated with cardiovascular complications, nephropathy and end stage-renal disease. Together with other elements of the metabolic syndrome, including hypertension, diabetes and obesity are interrelated and contribute to progression of renal disease. Multiple factors act in concert to initially cause renal vasodilation, glomerular hyperfiltration, and albuminuria, leading to the development of glomerulopathy [25–27]. Here we studied a model of diabetes and obesity in db/db mice, focusing on renal damage, and questioned whether statins ameliorate nephropathy, since our previous findings demonstrated significant vasoprotection by statins in these mice.

Major findings in the present study demonstrate that atorvastatin reduces renal hypertrophy and dysfunction in diabetic obese mice. These processes are associated with reduced ROS generation, downregulation of hypertrophic signaling pathways, decreased activation of redox-sensitive ERK1/2, and restoration of Akt and GSK3β activity. Protective actions of atorvastatin are not generalized phenomena, because renal inflammation in db/db mice was unaffected by statin treatment. Our data suggest that statins may have an important impact on diabetes-associated nephropathy. These effects are highly regulated and may be beneficial as adjuvant therapy in the renal complications of diabetes.

Statins, HMG-CoA reductase inhibitors, exhibit actions beyond their cholesterol-lowering properties, and seem to have direct vascular and renal protective effects, although exact mechanisms still remain unclear [5, 28–31]. We previously reported that atorvastatin improves endothelial function and ameliorates vascular inflammation through processes that reduce oxidative stress. Statins also influence antioxidant systems [28] and through formation of isoprenoids, promote hydrophobic modifications of small GTPases, such as Rac1/2, which play a crucial role in the activation of NADPH oxidases [28], and RhoA [30].

In the present study, atorvastatin reduced renal Nox4 protein expression and ROS generation and normalized RhoA activity in db/db mice. In addition, statins decreased diabetes-associated renal dysfunction (albuminuria) and prevented renal damage and cell proliferation, as evidenced d by PCNA protein expression and immunolocalization. In particular, db/db kidneys exhibited increased proliferation of mesangial and tubular epithelial cells p and hypertrophy, in line with previous studies [32,33]. Our data are in agreement with these and other studies showing that statins improve renal structure both in type 1 and type 2 experimental diabetes [8, 34–36] and also in patients with type 1 diabetes [37]. The effects of atorvastatin observed in our study do not seem to be agent-specific as other statins, such as simvastatin, pitavastatin and rosuvastatin have also been shown to have some renoprotective actions in experimental models of diabetes [8, 38].

Rho GTPases play an important role in renal integrity and function [29, 39, 40]. Of importance, transgenic mice expressing doxycycline-inducible constitutively active or dominant-negative RhoA in podocytes revealed that RhoA, in basal conditions, maintains the integrity of the glomerular filtration barrier, but increased RhoA activity promotes podocyte injury [39]. In addition, mice subjected to nephrectomy present podocyte dysfunction, which is attenuated by RhoA signaling inhibition [40]. RhoA also is involved in renal integrity in diabetic mice; its inhibitors attenuate diabetes-induced renal hypertrophy in different models of diabetes, including db/db mice [8, 41], supporting a role for RhoA/Rho kinase in diabetes-associated nephropathy. Of importance, inhibition of the small GTPase RhoA, by fasudil or statins, reduces renal Nox4 protein expression in streptozotocin-induced type 1 diabetes [1] as well as in db/db mice [35]. RhoA may be activated in the endoplasmic reticulum upstream of Nox4 in isolated endothelial cells [42]. TGF-β activates RhoA/ROCK signaling regulating Nox4 activity in kidney myofibroblasts [43]. These studies reinforce our findings suggesting that abnormalities in renal structure might be associated with altered RhoA signaling and Nox4-induced oxidative stress. Moreover, we found that RhoA content was increased in glomerular mesangial and tubular epithelial cells in db/db mice. Although atorvastatin did not significantly influence RhoA content, it reduced RhoA activity as evidenced by decreased cytosol-to-membrane translocation. Exact mechanisms whereby RhoA and Nox4 interact and how this impacts on podocyte dysfunction and mesangial proliferation remain elusive and warrant further investigation. Redox-sensitive proteins such as MAP kinases regulate many cellular functions including cell growth, differentiation, proliferation, and apoptosis [41]. Stress-activated protein kinase (SAPK)/JNK MAPK and p38MAPK are related mainly to stress or cell injury responses, while ERK1/2 is associated with mitogenic and growth factor stimulation [41, 42]. Whereas no differences were found in renal SAPK/JNK MAPK or p38MAPK phosphorylation/activity in kidneys of db/db mice, ERK1/2 activity was increased and may have contributed to diabetes-associated renal hypertrophy.

Other molecular processes underlying renal damage in diabetes include dysregulation of signaling pathways that control cell survival and cellular protection. In particular Akt is critical for podocyte survival and normal renal function [43]. An important downstream substrate of Akt is GSK3β [44]. In our study, both Akt and GSK3β were significantly downregulated in db/db mice and may reflect tissue damage and associated reduced cell survival. These findings are in line with the hypertophic and injured phenotype observed in the diabetic group in our study. Atorvastatin normalized activity of Akt and GSK3β, suggesting cellular protection, processes which may contribute to the overall renal protective actions of statin treatment in diabetes. Exactly how atorvastatin normalizes these processes is unclear, but may relate, in part, to reduced oxidative stress and decreased Rho kinase signaling. RhoA activation has been shown to trigger apoptosis dependent on increased phosphatase and tensin homolog (PTEN) activity, which inactivates the pro-survival protein Akt to suppress cell survival [45].

In conclusion, our data suggest that atorvastatin attenuates renal damage in diabetic mice through ROS- and RhoA-dependent mechanisms. Moreover, Akt and GSK3β normalization contribute to atorvastatin actions, processes that may be dependent on ERK1/2 activation. While atorvastatin improved renal functional and structural alterations in diabetes, inflammatory processes were not sensitive to statin treatment in our model. The present study advances our understanding on some molecular mechanisms related to type 2 diabetes-associated renal damage and identifies putative pathways modulated by statins. These findings have particular clinical relevance because recent studies have demonstrated that atorvastatin is renoprotective in patients with early diabetic nephropathy [37, 46]. The molecular mechanisms identified in our study may explain, at least in part, clinical benefits of atorvastatin in human diabetic kidney disease.
